# A new frameshift mutation of the β-spectrin gene associated with hereditary spherocytosis

**DOI:** 10.1007/s00277-016-2838-0

**Published:** 2016-10-06

**Authors:** Dżamila M. Bogusławska, Elżbieta Heger, Beata Machnicka, Michał Skulski, Kazimierz Kuliczkowski, Aleksander F. Sikorski

**Affiliations:** 1 Department of Molecular Biology, Faculty of Biological Sciences, University of Zielona Góra, Szafrana 1, Zielona Góra, 65-516 Poland; 2 Department of Haematology, Blood Neoplasms and Bone Marrow Transplantation, Wroclaw Medical University, Wybrzeże Pasteura 4, Wrocław, 50-367 Poland; 3 Department of Cytobiochemistry, Biotechnology Faculty, University of Wrocław, F. Joliot-Curie 14a, 50-383 Wrocław, Poland

Dear Editor,

Hereditary spherocytosis (HS) is the most frequent congenital haemolytic anaemia in Caucasians. β-spectrin defects are typically inherited in an autosomal dominant manner with the clinical presentation of HS [5, 9]. We report on a new mutation in the *SPTB* gene (466insG) leading to a frameshift and a premature stop codon 29 codons downstream in the region encoding the C-terminal part of the dimerization domain. Most probably, instability of mutant mRNA results in spectrin deficiency and clinically moderate to serious HS.

The N-terminal region of the β-spectrin subunit consists of actin-binding domain and located downstream the dimerization domain consisting of the first two spectrin repeats. During our studies on HS cases in the population of Western Poland, we found a new mutation in a family with autosomal dominant HS [2–4]. Patients with moderate symptoms of the disease were recruited: mother (C10, splenectomized) and children (C14, C9), age ranged from 27 to 67 years. Diagnostic criteria for HS were based on typical features: spherocytes on peripheral blood smears, increased osmotic fragility, splenomegaly, increased bilirubin, reticulocytosis, anaemia and symptoms of gallstone disease (Supplemental Table I). The Ethics Committee of the Medical University of Wroclaw approved the study protocol. Informed consent was obtained from all patients and healthy individuals serving as a control before entering the protocol.

Molecular genetic analyses of the studied patients and the unaffected family members were performed. The nucleotide sequence of the *ANK1*, *SPTB* and *SLC4A1* genes were amplified by PCR from genomic DNA. During DNA analysis of these genes, several common polymorphisms were encountered (Supplemental Table II). DNA sequencing revealed that all of the studied patients were heterozygous for novel mutation 466insG in the exon 11 of the *SPTB* gene (Fig. 1a, b).

**Fig. 1:**
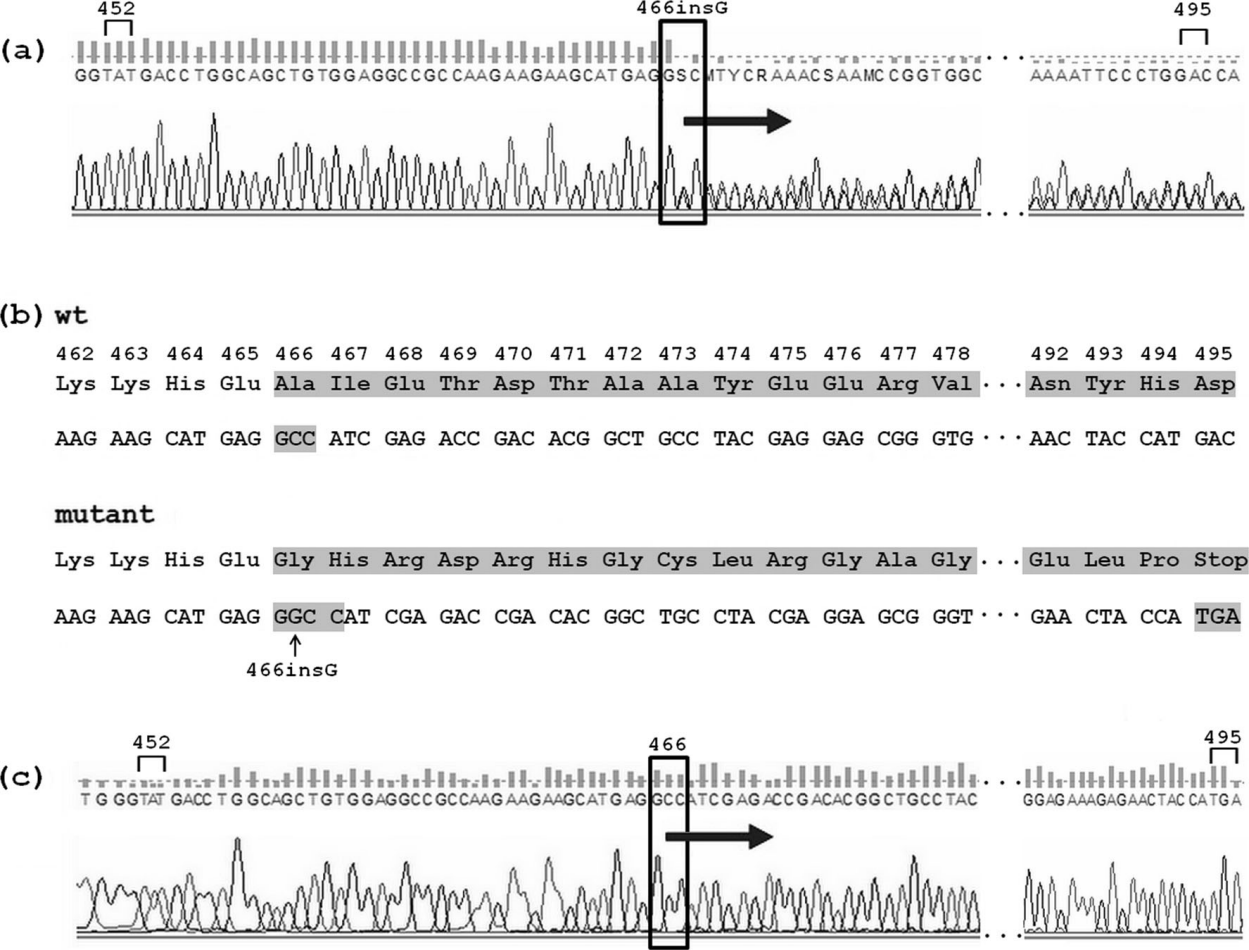
Fragment of sequencing traces detected in the patient C14 showing: **a** A heterozygous single nucleotide 466insG in the exon 11 of the *SPTB* gene (genomic DNA was used as a template). **b** 466insG leading to a frameshift and a premature stop codon 29 codons downstream. **c** Loss of mutant allele in the cDNA in relation to the genomic DNA (cDNA was used as a template)

RBC membrane protein gel electrophoresis demonstrated deficiency of some membrane proteins: β-spectrin and ankyrin-1, thereby supporting the diagnosis of HS (data not shown). Immunoblotting of erythrocyte membrane ghosts from C14 and C10 patients with this mutation with antibody recognising an actin binding domain of β-spectrin [10] could not detect a truncated protein (data not shown), suggesting that this mutant transcript might be unstable.

Next, we assessed the presence of the mutant mRNA transcripts. Loss of mutant allele in the cDNA in relation to the genomic DNA was observed (Fig. 1c), indicating that the mutant mRNA transcript was not present to detectable levels. Frameshift mutations of the *SPTB* gene associated with HS have been previously reported and in some cases the mutant mRNA could not be detected or was detected in decreased levels [1, 6–8]. Also, the truncated protein could be observed in the erythrocyte or reticulocyte membrane [7]. As in the studied family the presence of mutated mRNA or truncated protein was not observed in the reticulocytes/erythrocytes, we conclude that in the studied family members probably monoallelic expression of the *SPTB* gene takes place which results in spectrin deficiency. This in consequence is a reason for a decreased surface density of the membrane skeleton and disturbed support of the membrane lipid bilayer and hereditary spherocytosis.

## Electronic supplementary material

Below is the link to the electronic supplementary material.

ESM 1 (DOCX 21 kb)
