# Supplementary material for: A new frameshift mutation of the β-spectrin gene associated with hereditary spherocytosis
Source: Ann Hematol. 2016 Oct 6;96(1):163–5. doi: 10.1007/s00277-016-2838-0 (PMC5203818; doi:10.1007/s00277-016-2838-0)
Supplement: Supplementary file 1 — (DOCX 21 kb) [file 277_2016_2838_MOESM1_ESM.docx]

**Supplemental Table I** Hematological data of the studied HS patients with a novel *SPTB* gene mutation.

| **HS patients** | **Age (years)** | **RBC  [*g/l*]** | **HCT**  **[*%*]** | **Hb**  **[*g/l*] *(remark)*** | **Total bilirubin**  **[*mg/dl*]** | **Ret**  **[*%*]** | **Spherocytes  in a peripheral blood smear** | **Direct**  **antiglobulin test (DAT)** | **EMA test**  **[*%*]** |
| --- | --- | --- | --- | --- | --- | --- | --- | --- | --- |
| C10 mother (female)  before splenectomy | 67 | 1.67-3.60 | 12-28 | 45-99 | 1.57-2.90 | 5.2-19.6 | few | negative | n.d. |
| C10 mother (female)  after splenectomy |  | 4.40-5.23 | 39-46 | 123-159 | 0.53-0.80 | 1.9-3.4 | numerous |  |  |
| C9 daughter (female) not splenectomied | 28 | 3.31 | 26 | 95 | 3.52 | 9.5 | few | negative | 70,3 |
| C14 son  (male)  not splenectomied | 39 | 3.40-4.50 | 32-38 | 110-134 | 1.85-4.30 | 7.8-14.5 | few | negative | 70,2 |

**Supplemental Table II** Polymorphisms identified during the sequence analysis of all of the tested genes in the subjects from the studied family.

| **Location in gene** | **Type of sequence change** | **Chromosome position** | **Nucleotide change (SNP to Chr)** | **Change of amino acid residue** | **Inheritance** | **SNP reference number** |
| --- | --- | --- | --- | --- | --- | --- |
| ***ANK1*** *(Homo sapiens* chromosome 8 genomic contig - NT_167187.2) | | | | | | |
| Intron 4 | Substitution | 41728053 | G→A (REV) | - | Heterozygotic | rs1872877 |
| Exon 5 | Substitution | 41727920 | T→C (REV) | N105N | Heterozygotic | rs2304871 |
| Intron 10 | Substitution | 41723089 | T→C (FWD) | - | Heterozygotic | rs7826127 |
| Exon 26 | Substitution | 41696410 | G→C (REV) | L971L | Heterozygotic | rs504574 |
| Exon 39 | Substitution | 41668396 | T→C (FWD) | V1755V | Heterozygotic | rs750625 |
| Exon 41  (nearGene-5) | Substitution | 41661730 | G→A (REV) | - | Homozygotic | rs516946 |
| Exon 42  (UTR-3) | Short tandem repeat (microsatellite variation) | 41655131- -41655132 | (TG)13/15 (FWD) | **-** | Heterozygotic | rs3138830 |
| Exon 42  (UTR-3) | 14-nt Deletion | 41655068- -41655081 | del AGAGTCTATACAGC (FWD) | **-** | Homozygotic | rs57963234 |
| ***SPTB*** *(Homo sapiens* chromosome 14 genomic contig - NT_026437.13) | | | | | | |
| Intron 10 | Substitution | 64795707 | T→G (REV) | **-** | Heterozygotic | rs229589 |
| ***SLC4A1*** *(Homo sapiens* chromosome 17 genomic contig - NT_010783.16) | | | | | | |
| Intron 3 | Substitution | 44262581 | G→T (FWD) | - | Heterozygotic | rs45469892 |
| Intron 13 | Substitution | 44257582 | A→G (REV) | - | Homozygotic | rs2252501 |
| Intron 15 | Substitution | 44255435 | C→T (FWD) | - | Heterozygotic | rs11870606 |
